# 

**Published:** 1871-09

**Authors:** 


*American Academy of Dental Science.*—The Fourth Annual Meeting of the American Academy of Dental Science, will be held in Boston on Monday, September, 25th, at eleven o'clock, A. M.

The Annual Address will be delivered by Professor J. H. McQuillen, of Philadelphia.

